# Isoscattering non-isospectral quantum graphs

**DOI:** 10.1038/s41598-025-23400-5

**Published:** 2025-11-12

**Authors:** Omer Farooq, Michał Ławniczak, Pavel Kurasov, Leszek Sirko

**Affiliations:** 1 Institute of Physics, Polish Academy of Sciences, Aleja Lotników 32/46, 02-668 Warszawa, Poland; 2 Department of Mathematics, Stockholm University, S-106 91 Stockholm, Sweden

**Keywords:** Mathematics and computing, Physics

## Abstract

Using the formalism of the Titchmarsh-Weyl M-function we study a pair of isoscattering non-isospectral quantum graphs. These graphs have the same total length *L*, but different topologies. The isoscattering but non-isospectral quantum graphs are difficult to identify and very complex to analyze. However, failing to recognize their existence will lead to incorrect interpretation of the spectra. We show that a potential experimental solution to this problem could involve constructing a pair of parameter-dependent non-isoscattering and non-isospectral graphs with the same total length *L*. Subsequently, by implementing the transition of the parameter towards zero, it can be demonstrated that the graphs transform into a pair of isoscattering graphs that remain non-isospectral. These theoretical predictions are subjected to experimental verification using microwave networks.

## Introduction

Quantum graphs are mathematical one-dimensional constructions of networks consisting of edges joined by vertices. The idea of such systems was introduced by Linus Pauling^1^ to study free electrons in organic molecules. Subsequently, this concept was employed in a variety of applications, encompassing quantum circuits, photonic crystals, microelectronics, theories concerning superfluidity and microwave networks^2–13^.

The study of isospectral systems was initiated by the seminal question posed by Mark Kac (1966): “Can you hear the shape of a drum?”, which addressed the issue of distinguishing Laplace spectra on planar domains. The isospectral Riemannian manifolds were described by Sunada^14^ in 1985. A few years later, pairs of planar isospectral domains were also created^15–18^. This definitively answered Kac’s question in the negative.

A similar question was later extended to quantum graphs^20^ and microwave networks^13^ through its modified version “Can one hear the shape of a graph?”

Kotani and Sunada’s seminal work^19^ was the first to investigate the role of graph symmetry on its spectral properties. They explored how symmetry-breaking in quantum graphs could lead to isospectral graphs. Subsequently, it was demonstrated in^20,21^ that incommensurate bond lengths of a graph uniquely determine its structure. However, upon relaxing this constraint, it was revealed that there exist isospectral graphs with different structures. Furthermore, the systematic methods for constructing isospectral quantum graphs were provided in Refs.^22–26^.

An important aspect of quantum graphs is the interlink between isospectrality and isoscattering^27,28^.

Practical applications of isoscatttering and isospectral graphs includes designing structures with specific vibration properties in acoustics and engineering^29^, as well as potential uses in secure communication and cryptography^30^. Additionally, experimental studies using microwave networks^13,31,32^ and optical systems help validate spectral theories and develop new materials with tailored properties^34^.

Isoscattering non-isospectral graphs present a significant challenge to conventional assumptions in spectral graph theory. The study of such graphs has the potential to facilitate a more profound comprehension of wave behavior in complex systems, the relationship between geometry and spectral properties, and their implementation in various applications.

Therefore, in this study, we construct a unique pair of isoscattering non-isospectral graphs and investigate their properties.

## M-functions and the scattering matrix

### Metric graphs and differential operators

A metric graph $\Gamma = (V, E)$ consists of a finite set of edges $E = \{e_n\}$, each with a finite length $l_n$, connected at the edges $V = \{ v_m \}$. The topological structure of $\Gamma$ is characterized by the connectivity between its edges, while geometric properties are determined by the edge lengths. Each vertex $v_i$ has a valency $d_i$, indicating the number of edges attached to it. Each edge $e_n$ is associated with a compact interval on a separate copy of the real line, expressed as $e_n = [x_{2n-1}, x_{2n}]$. A metric graph turns into a quantum graph when a Schrödinger differential operator is imposed on its edges. Quantum graphs serve as effective models for quantum chaos, mesoscopic physics, and wave propagation in disordered systems. One of the most fundamental operators is the Laplacian, defined by the second derivative operator $L(\Gamma ) = -\frac{d^2}{dx^2}$, which acts on functions defined along the edges of the graph and satisfying the standard (Kirchhoff-Neumann) conditions, given by

1 $$\begin{aligned} \left\{ \begin{array}{ll} \displaystyle u(x_i)=u(x_j), & x_i, x_j \in v_m, \\ \displaystyle \sum _{x_i{ \in v_m} }\partial _{n}u(x_i)=0, & \end{array} \right. \end{aligned}$$

where $u(x_i)$ and $\partial u (x_i)$ are limiting values along the edges at their endpoints of the functions and their first derivatives taken in the direction inside the edges. These conditions enforce the continuity of the wave function not only across edges but at the vertices as well and ensure the conservation of probability current at each vertex^4,5^.

The role of vertex conditions is two-fold: they connect the wave functions on different edges and therefore determine the wave dynamics on the metric graph, they are needed to make the differential operator self-adjoint and therefore suitable to be used as a quantum mechanical model.

The above vertex conditions correspond to the simplest quadratic form given by the Dirichlet integral $\int _\Gamma \vert u'(x) \vert ^2 dx$ defined on continuous at vertices functions, hence the name standard. One usually refers to the spectrum of this Laplacian as the spectrum of the metric graph $\Gamma$.

In general, if we assume that the functions are continuous at the vertices, then all possible local vertex conditions that guarantee flux preservation are $\delta$ couplings (see for example formula (3.59) in^5^). It is customary to interpret non-zero couplings as $\delta$ potentials at the vertices. Zero couplings correspond to standard vertex conditions which we use throughout the paper.

The Laplacian operator $L(\Gamma )$ exhibits a discrete, non-negative spectrum $\lambda _1 < \lambda _2 \le \lambda _3 \le \dots .$ The ground state is non-degenerate in the case of connected graphs and is spanned by the constant function. The spectrum of $L(\Gamma )$ is highly sensitive to the topology and geometry of the metric graph, *i.e.* to connections between the edges and their lengths, respectively. If the graphs are equilateral (have the same or pairwise rationally dependent edge lengths), then the spectrum is periodic in the *k*-scale, $k^2 = \lambda$. Another extreme case when the edge lengths are incommensurate, *i.e.* rationally independent, is especially interesting since the spectrum does not contain non-trivial infinite arithmetic progressions^35^.

### Scattering on compact graphs

With any (compact) metric graph $\Gamma$ we may associate a scattering matrix $S_{\Gamma }(k)$, provided a finite set $\partial \Gamma$ of contact vertices is specified. Let us construct another (non-compact) graph $\Gamma ^\textrm{ext}$ obtained by attaching to $\Gamma$ precisely $| \partial \Gamma |$ infinite leads - the semi-infinite intervals parametrized as $[0, \infty )$. We assume that standard vertex conditions are introduced in the new vertices obtained by adding left end-points of the semi-infinite intervals to each of the contact vertices $\partial \Gamma$.

Consider any solution of the eigenfunction equation

$$- u'' (x) = k^2 u(x)$$

on the edges satisfying vertex conditions at all vertices. For $k^2 >0$, where *k* is a wave vector, these solutions are combinations of plane waves on the non-compact edges:

$$u(x) = a_n e^{-i kx} + b_n e^{ikx}, \quad x \in e_N, \quad e_n \in \Gamma ^\textrm{ext} \setminus \Gamma .$$

The corresponding stationary scattering matrix $S_{\Gamma }(k)$ connects the amplitudes of the plane waves:

2 $$\begin{aligned} S_{\Gamma }(k) \vec {a} = \vec {b}, \end{aligned}$$

where the vectors $\vec {a}, \vec {b}$ have dimension $|\partial \Gamma |$ and correspond to semi-infinite edges.

### M-functions

The Titchmarsh-Weyl M-functions form a powerful tool in the spectral theory of one-dimensional Schrödinger operators^5^, Ch. 17. The M-function captures important spectral information and therefore can be used to describe (part of) graph’s spectra.

We define the M-function associated with the compact graph $\Gamma$ and a set of contact vertices $\partial \Gamma$ as

3 $$\begin{aligned} M_\Gamma (k) : \vec {u}_{\partial \Gamma } \mapsto \partial \vec {u}_{\partial \Gamma } \end{aligned}$$

where $\vec {u}_{\partial \Gamma }, \partial \vec {u}_{\partial \Gamma }$ denote the values of the function and the sums of normal derivatives at the vertices from the contact set $\partial \Gamma$ of any solution *u* of the eigenfunction equation $-u''=k^2u$ on the metric graph, satisfying standard conditions at all non-contact vertices and just continuity conditions at the contact vertices. (Note that the definition of the M-function does not involve the extended graph $\Gamma ^\textrm{ext}$ used in the definition of the scattering matrix.)

In case the contact set consists of a single vertex *v* the definition can be simplified:

4 $$\begin{aligned} M_\Gamma (k) = \frac{\partial u (v)}{u (v)}. \end{aligned}$$

For any metric graph, $\Gamma$ the formula connecting the S-matrix and the M-matrix reads as follows (formula (18:40) in^5^)

5 $$\begin{aligned} S_\Gamma (k) = \frac{ ikI - M_\Gamma (k)}{ikI + M_\Gamma (k)}, \end{aligned}$$

where *I* is the unity matrix. This formula determines $| \partial \Gamma | \times | \partial \Gamma |$ matrix valued function. Since any dissipation-free metric graph $\Gamma$, $M_\Gamma (k)$ is Hermitian almost everywhere, $S_\Gamma (k)$ is unitary.

With the graph, $\Gamma$ we may associate two self-adjoint operators with discrete spectrum:

1. the operator $L^\textrm{st}(\Gamma )$ determined by standard vertex conditions on $\partial \Gamma$ (and any Hermitian conditions at the other vertices);
2. the operator $L^\textrm{D}(\Gamma )$ determined by Dirichlet conditions on $\partial \Gamma$ (and any Hermitian conditions at the other vertices).

Then zeroes and singularities of the M-matrix correspond to the eigenvalues of these operators denoted by $( k_n^\textrm{st})^2$ and $(k_n^\textrm{D})^2$ respectively. Note that in general not all eigenvalues of $L^\textrm{st}$ and $L^\textrm{D}$ are visible from the M-function. For example all common eigenvalues of these operators having common eigenfunctions are not seen in the M-function.

### Isoscattering and isospectrality

In general two metric graphs $\Gamma _1$ and $\Gamma _2$ with contact sets $\partial \Gamma _1$ and $\partial \Gamma _2$, respectively, are isoscattering if there exists a transplantation matrix *T*^31,32^ independent of *k* which satisfies:

6 $$\begin{aligned} S_{\Gamma _2} (k) = {T S}_{\Gamma _1}(k) T^{-1}. \end{aligned}$$

In particular isoscattering graphs must have the same number of contact vertices $| \partial \Gamma _1 | = | \partial \Gamma _2 |.$

In this paper we consider the simplest case of a single contact vertex. Note that the scattering matrix is scalar, this leads to the isoscattering condition: $S_{\Gamma _2} (k) = S_{\Gamma _1}(k)$. Formula (5) implies that two graphs $\Gamma _1$ and $\Gamma _2$ are isoscattering if the corresponding M-functions are equal $M_{\Gamma _2} (k) = {M}_{\Gamma _1}(k)$. Therefore, the M-function formalism is a powerful framework for describing isoscattering graphs.

Identical M-functions do not imply that the corresponding graphs are isospectral due to possible presence of invisible eigenvalues, whose eigenfunctions satisfy both Dirichlet and Kirchhoff-Neumann conditions at the contact vertices. These eigenfunctions do not contribute to M-function and therefore cannot be detected (see formulas (17.26) and (17.37) in^5^). Some of these eigenfunctions are supported by edges forming loops and the eigenvalues depend continuously on the loop lengths. The other invisible eigenvalues may be sensitive to changes in the other edges - they turn immediately into resonances when the lengths of the edges supporting the corresponding eigenfunctions are changed.

**Fig. 1:**
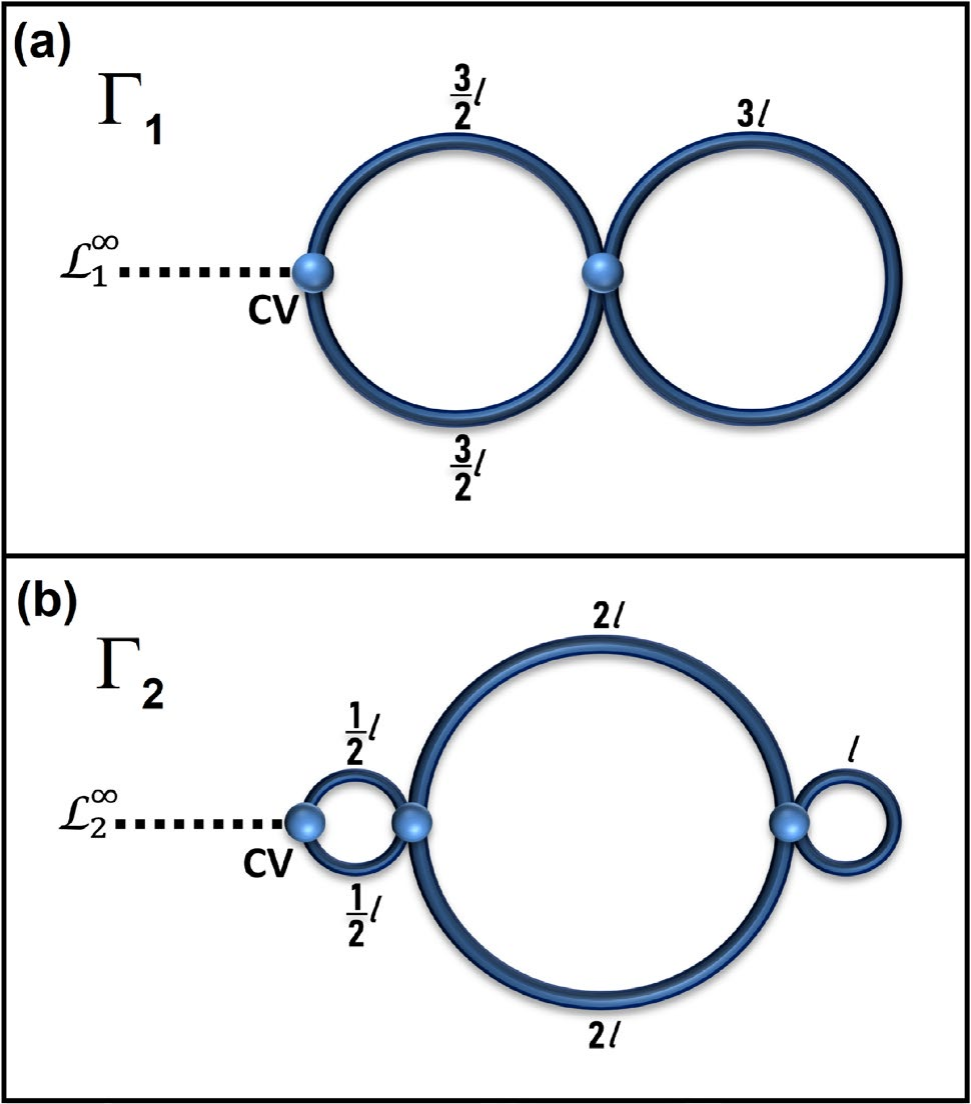
Panels (**a**) and (**b**) show graphs $\Gamma _1$ and $\Gamma _2$ with standard boundary conditions at the vertices. The graphs possess the same total length $L=6l=2.247 \pm 0.003$ m. The contact vertices are labeled by *CV*. In the experimental realization of the graphs, the microwave networks are connected to a microwave vector network analyzer via leads $\mathscr {L}^{\infty }_1$ and $\mathscr {L}^{\infty }_2$, respectively.

We will use this property to demonstrate that analyzed by us graphs $\Gamma _1$ and $\Gamma _2$ (see Fig. 1), despite being isoscattering, are non-isospectral. This pair of graphs is a generalization of isoscattering but non-isospectral graphs presented in^33^. Our main focus is on demonstrating how non-isospectrality of these graphs can be observed experimentally.

**Fig. 2:**
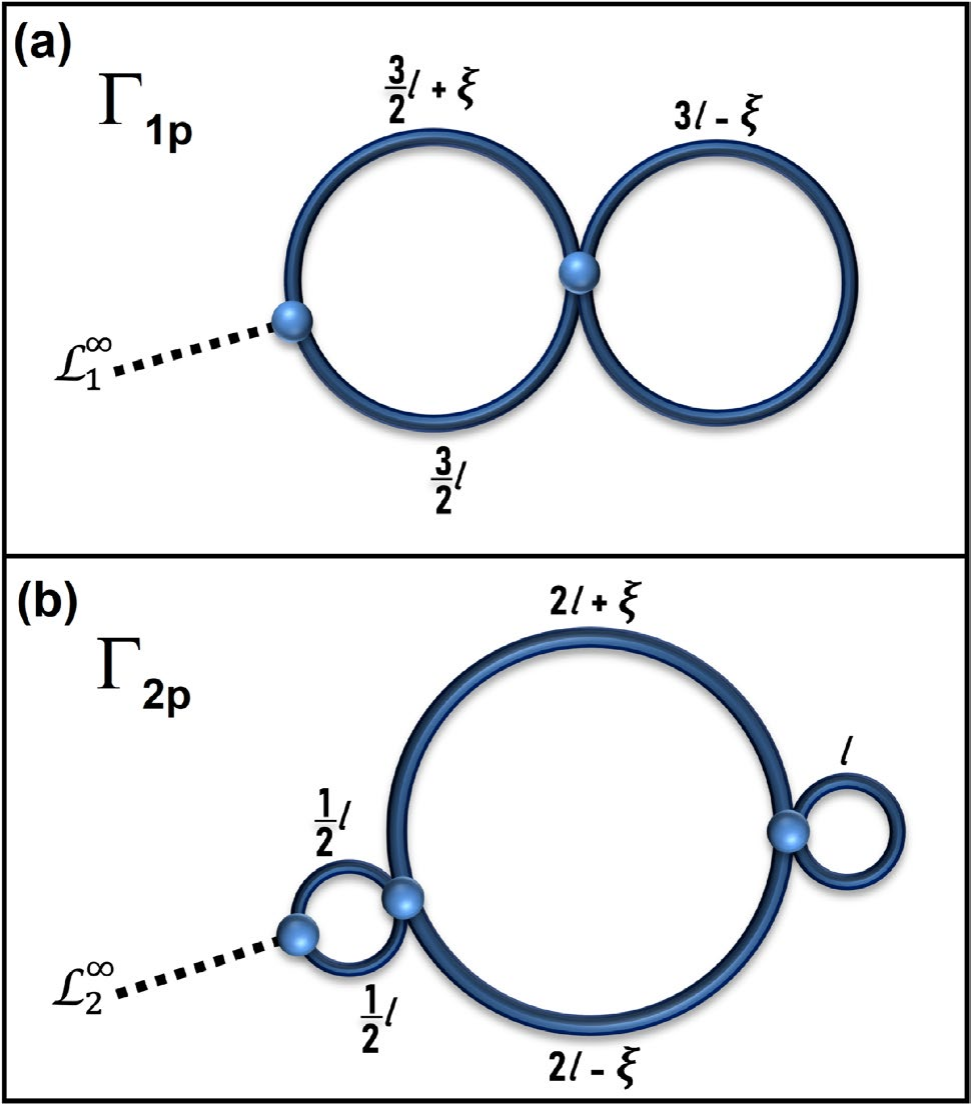
Panels (**a**) and (**b**) show perturbed graphs $\Gamma _{1p}$ and $\Gamma _{2p}$ with standard boundary conditions at the vertices. The graphs are characterized by the same total length $L=6l=2.247 \pm 0.003$ m. The perturbation parameter is denoted by $\xi$. In the experimental investigation of graphs $\Gamma _{1p}$ and $\Gamma _{2p}$, the parameter $\xi$ was chosen to be 0.02 m. The networks are connected to a microwave vector network analyzer via leads $\mathscr {L}^{\infty }_1$ and $\mathscr {L}^{\infty }_2$, respectively.

We will begin our analysis with studying the graphs $\Gamma _{1p}$ and $\Gamma _{2p}$, presented in Fig. 2. These graphs are dependent on the parameter $\xi$ and are neither isoscattering, nor isospectral. We show that, in the limit $\xi \rightarrow 0$, graphs $\Gamma _{1p} \rightarrow \Gamma _1$ and $\Gamma _{2p} \rightarrow \Gamma _2$ become isoscattering, but remain non-isospectral.

To calculate the corresponding M-functions we consider first the M-functions in the case when all vertices are contact vertices. From this M-function it is relatively easy to calculate the M-functions associated with a single contact vertex by reduction as explained in Section 17.3 of^5^. Formula (17.44) is of particular importance for us.

The graph $\Gamma _{1p}$ in Fig. 2a has two vertices and the corresponding $2 \times 2$ M-function is

$$\textbf{M}(k) = k \left( \begin{array}{cc} - \cot k (3\ell /2+\xi ) - \cot k (3\ell /2) & \frac{1}{\sin (3 \ell /2+\xi ) }+ \frac{1}{\sin (3 \ell /2) }\\[3mm] \frac{1}{\sin (3 \ell /2+\xi )} + \frac{1}{\sin (3 \ell /2) } & - \cot k (3\ell /2+\xi ) - \cot k (3\ell /2) \\ & - 2 \cot k (3 \ell -\xi ) + \frac{2}{\sin k (3 \ell -\xi ) } \end{array} \right) .$$

Taking into account standard conditions at the right (internal) vertex we get the following expression for the M-function associated with the single (left) contact vertex:

7 $$\begin{aligned} \begin{array}{ccl} M_{\Gamma _{1p}} (k) & = & \frac{1}{M_{22}} \left( M_{11} M_{22} - M_{12} M_{21} \right) \\ & =& k \frac{ (- \cot k (3\ell /2+\xi ) - \cot k (3\ell /2- \xi )) ( - \cot k (3\ell /2+\xi ) - \cot k (3\ell /2- \xi ) - 2 \cot k 3 \ell + \frac{2}{\sin k 3 \ell }) - \left( \frac{1}{\sin k (3 \ell /2+\xi ) }+ \frac{1}{\sin k (3 \ell /2-\xi ) } \right) ^2}{- \cot k (3\ell /2+\xi ) - \cot k (3\ell /2- \xi ) - 2 \cot k 3 \ell + \frac{2}{\sin k 3 \ell }}. \end{array} \end{aligned}$$

The second graph $\Gamma _{2p}$ in Fig. 2b has three vertices. The $3 \times 3$ M-function associated with all vertices is given by

$$\textbf{M}(k) = k \left( \begin{array}{ccc} - 2 \cot k \ell /2 & \frac{2}{\sin k \ell /2} & 0 \\ \frac{2}{\sin k \ell /2} & - 2 \cot k \ell /2 - \cot k (2 \ell + \xi ) & \frac{1}{\sin k (2 \ell +\xi )} + \frac{1}{\sin k (2\ell -\xi )} \\ & - \cot k (2 \ell -\xi ) & \\ 0 & \frac{1}{\sin k (2 \ell +\xi )} + \frac{1}{\sin k (2\ell -\xi )} & - \cot k (2 \ell + \xi ) - \cot k (2 \ell -\xi ) \\ & & - 2 \cot k \ell + \frac{2}{\sin k \ell } \end{array} \right) .$$

Taking into account standard conditions at the inner vertices we get analog of formula (17.44)

$$M_{\Gamma _{2p}} (k) = M_{11} - M_{12} \frac{M_{33}}{M_{22} M_{33} - M_{23} M_{32}} M_{21}$$

leading to the following formula for the M-function associated with the single (left) contact vertex

8 $$\begin{aligned} \begin{array}{ccl} M_{\Gamma _{2p}} (k) & = & - 2 k\cot k \ell /2 - k\left( \frac{2}{\sin k \ell /2} \right) ^2 \\ & & \frac{ - \cot k (2 \ell + \xi ) - \cot k (2 \ell -\xi ) - 2 \cot k \ell + \frac{2}{\sin k \ell }}{ ( - 2 \cot k \ell /2 - \cot k (2 \ell + \xi ) - \cot k (2 \ell -\xi ))( - \cot k (2 \ell + \xi ) - \cot k (2 \ell -\xi ) - 2 \cot k \ell + \frac{2}{\sin k \ell }) - \left( \frac{1}{\sin k (2 \ell +\xi )} + \frac{1}{\sin k (2\ell -\xi )} \right) ^2}. \\ \end{array} \end{aligned}$$

### Isoscattering but non-isospectral graphs

In Fig. 1 we show graphs $\Gamma _1 \equiv \Gamma _{1p} \vert _{\xi = 0}$ and $\Gamma _2 \equiv \Gamma _{2p} \vert _{\xi = 0}$. Using formulas (7) and (8) one can easily show that for $\xi =0$ both graphs have the same M-functions associated with the contact vertices CV:

9 $$\begin{aligned} M_{\Gamma _1} (k) = k \frac{ - 2\cot k 3\ell /2 ( -2 \cot k 3\ell /2 - 2 \cot k 3 \ell + \frac{2}{\sin k 3 \ell }) - \left( \frac{2}{\sin k 3 \ell /2 } \right) ^2}{- 2 \cot k 3\ell /2 - 2 \cot k 3 \ell + \frac{2}{\sin k 3 \ell }} = 2k \tan 3 k \ell . \end{aligned}$$

and

10 $$\begin{aligned} \begin{array}{ccl} M_{\Gamma _2} (k) & = & - 2k \cot k \ell /2 - k\left( \frac{2}{\sin k \ell /2} \right) ^2 \frac{ - 2\cot k (2 \ell ) - 2 \cot k \ell + \frac{2}{\sin k \ell }}{ ( - 2 \cot k \ell /2 - 2\cot k (2 \ell ) )( - 2\cot k (2 \ell ) - 2 \cot k \ell + \frac{2}{\sin k \ell }) - \left( \frac{2}{\sin k (2 \ell )} \right) ^2} \\ & = & 2k \tan 3 k \ell . \end{array} \end{aligned}$$

Since

11 $$\begin{aligned} M_{\Gamma _1} (k) \equiv M_{\Gamma _2} (k) = 2 k \tan 3 k \ell , \end{aligned}$$

both graphs are isoscattering, as follows from Eq. (5).

Although the graphs are isoscattering, they are not isospectral. The spectra $\sigma _{\Gamma _1}(k)$ and $\sigma _{\Gamma _2}(k)$ of the dissipation-free graphs $\Gamma _1$ and $\Gamma _2$ are given by:

$$\begin{array}{*{20}c} {\sigma _{{\Gamma _{1} }} (k)} & = & {\frac{\pi }{{6\ell }}\{ 0,2,4,4,4,6,8,8,8,10,12,12,12,14,15 \ldots \} ,} \\ {} & {} & {} \\ {\sigma _{{\Gamma _{2} }} (k)} & = & {\frac{\pi }{{6\ell }}\{ 0,2,3,4,6,6,8,9,10,12,12,12,12,14,15 \ldots \} .} \\ \end{array}$$

To see directly that the two graphs are not isospectral, let us note that the metric graphs have horizontal symmetry axis. Hence all eigenfunctions can be divided into even and odd with respect to this symmetry. The even eigenfunctions on the two graphs can be identified, the corresponding spectra coincide, let us therefore turn to odd eigenfunctions. The graph $\Gamma _1$ has eigenfunctions given by $\sin$ function supported by the left loop, the lowest corresponding eigenvalue is $\left( \frac{2 \pi }{3\ell } \right)$. But this value of *k* is not an eigenvalue of the second graph corresponding to any odd eigenfunction: any such eigenfunction should be equal to zero at the vertices, which is impossible.

The existence of isoscattering but non-isospectral graphs poses a significant challenge to the measurement of spectra, since the differences between them are obscured by the properties of the scattering matrices. For example, for low frequency range the spectrum $\sigma _{\Gamma _2}(k)$ contains non-degenerate eigenvalues $k_3=\frac{\pi }{2\ell }$ and $k_8=\frac{3\pi }{2\ell }$, which are well separated from the eigenvalues of the spectrum $\sigma _{\Gamma _1}(k)$. However, these eigenvalues, which are resonances in an open microwave network, are not visible in the amplitude or phase of the scattering matrix.

Therefore, to demonstrate experimentally that the graphs $\Gamma _1$ and $\Gamma _2$ are non-isospectral, the scattering matrices of the graphs $\Gamma _{1p}$ and $\Gamma _{2p}$ (see Fig. 2) should be evaluated for small values of the parameter $\xi$. Departure of the scattering matrices from each other for $\xi \rightarrow 0$ will indicate the non-isospectrality ($\sigma _{\Gamma _{1p}}(k) \ne \sigma _{\Gamma _{2p}}(k)$) of the graphs $\Gamma _1$ and $\Gamma _2$. This will be discussed in detail in Section III. Experimental Observations.

## Experimental observations

Microwave networks are a powerful experimental tool for studying properties of quantum graphs and helping to understand quantum chaos and complex phenomena. This is due to the equivalence between the one-dimensional Schrödinger equation, describing quantum graphs, and the telegraph equation that describes microwave networks^2^. Microwave networks can model a variety of quantum graphs, including those characterized by the principal ensembles of Random Matrix Theory: Gaussian Orthogonal Ensembles^2,36–38,40–42^, Gaussian Unitary Ensembles^2,43–49^ and Gaussian Symplectic Ensembles^49–51^. In practice, quantum graphs can be simulated by constructing microwave networks consisting of coaxial cables (edges $\{e_{i}\}$), and microwave junctions (vertices $\{v_{i}\}$), e.g. T-junctions and 4-way junctions. Each vertex in the network is linked to the other vertices by edges, forming the same structure and topology as a quantum graph^3^ the properties of which should be investigated. The network’s scattering properties can be measured using a vector network analyzer (VNA)^36^. As with quantum graphs, the boundary conditions at the vertices significantly influence the spectral and scattering properties of the networks^38^. For instance, the Kirchhoff-Neumann boundary conditions impose the continuity of waves and vanishing of the sum of outgoing derivatives at each vertex $v_{i}$.

**Fig. 3:**
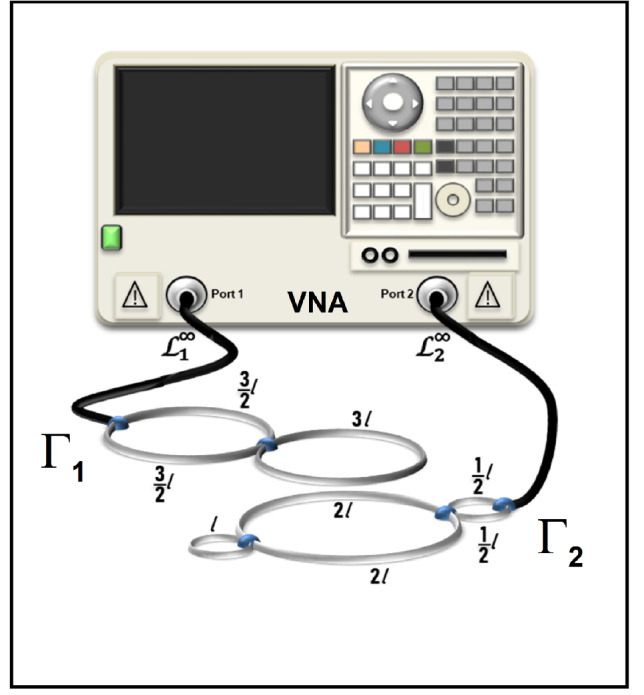
A scheme of the experimental set-up. Microwave networks $\Gamma _1$ and $\Gamma _2$ are subject to standard boundary conditions. Both networks are characterized by the same total length $L=6l=2.247 \pm 0.003$ m. The networks are connected at the contact vertices to a microwave vector network analyzer (VNA) via microwave cables (leads) $\mathscr {L}^{\infty }_1$ and $\mathscr {L}^{\infty }_2$, respectively.

**Fig. 4:**
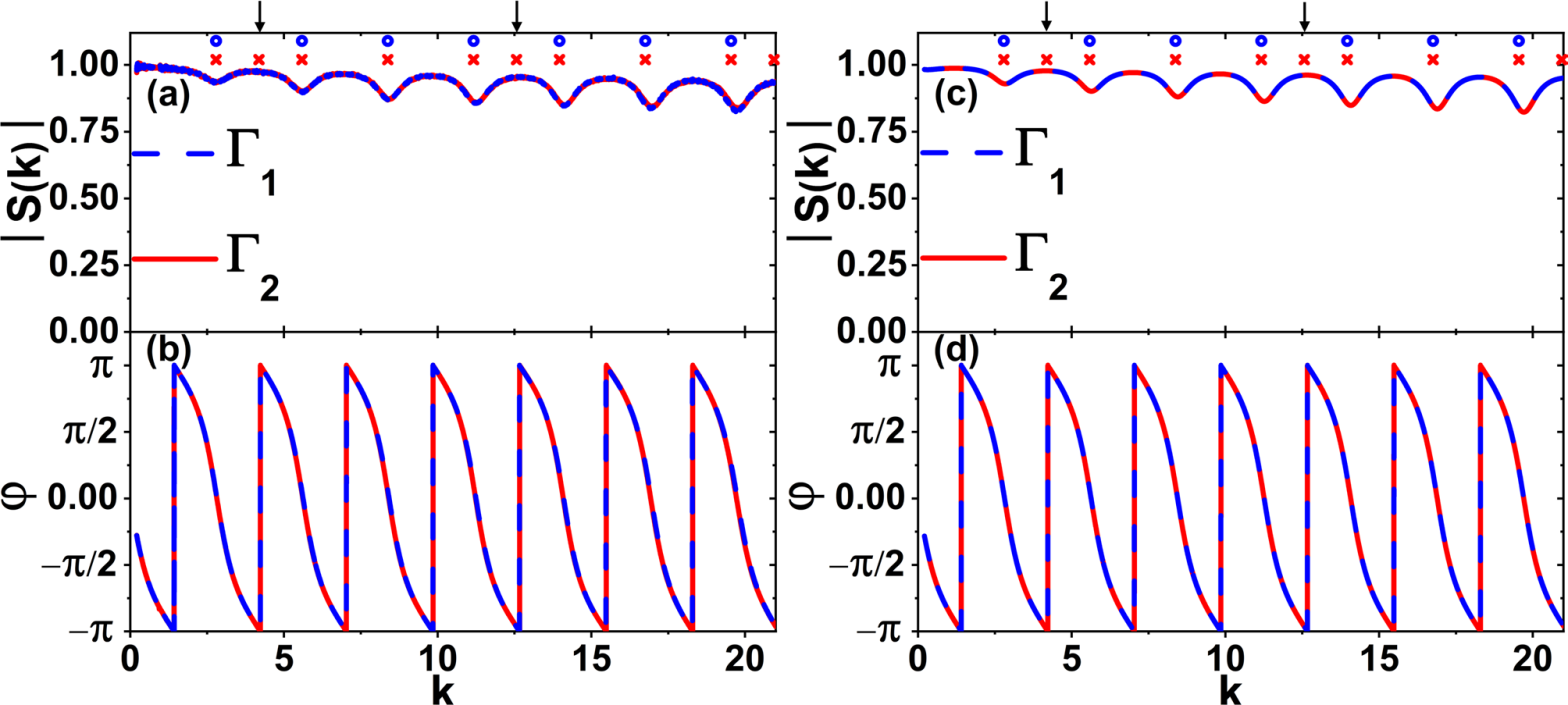
Panels (**a**) and (**b**) show the amplitudes |*S*(*k*)| and the phases $\varphi$ of the scattering matrices *S*(*k*) of the experimentally studied microwave networks $\Gamma _{1}$ (blue broken line) and $\Gamma _{2}$ (red solid line) in the frequency range $0.01-1$ GHz ($k=0.21-20.96$ $\frac{1}{m}$). The positions of the resonances $\sigma _{\Gamma _1}(k)$ and $\sigma _{\Gamma _2}(k)$ are marked by blue circles and red crosses, respectively. Singular resonances of the dissipation-free graph $\Gamma _{2}$, $k_3 = \frac{\pi }{2\ell } \simeq 4.19$ $\frac{1}{m}$ and $k_8 = \frac{3\pi }{2\ell } \simeq 12.58$ $\frac{1}{m}$, are additionally indicated by black arrows. (**c**) The absolute value |*S*(*k*)| and (**d**) the phase $\varphi$ of the scattering matrices *S*(*k*) calculated for the quantum graphs with dissipation emulating microwave networks $\Gamma _{1}$ (blue broken line) and $\Gamma _{2}$ (red solid line), respectively. In the calculations Eqs. (11) and (5) were applied.

**Fig. 5:**
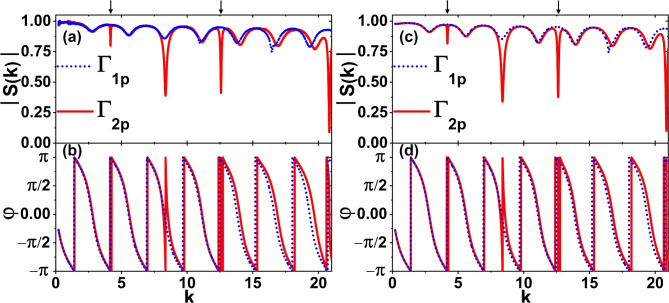
Panels (**a**) and (**b**) show the amplitudes |*S*(*k*)| and the phases $\varphi$ of the scattering matrices *S*(*k*) of the experimentally studied perturbed microwave networks $\Gamma _{1p}$ (blue dotted line) and $\Gamma _{2p}$ (red solid line) in the frequency range $0.01-1$ GHz ($k=0.21-20.96$ $\frac{1}{m}$). Singular resonances of the network $\Gamma _{2p}$, $k_3 \simeq 4.19$ $\frac{1}{m}$ and $k_8 \simeq 12.60$ $\frac{1}{m}$, are marked by black arrows. (**c**) The absolute value |*S*(*k*)| and (**d**) the phase $\varphi$ of the scattering matrices *S*(*k*) calculated for the quantum graphs with dissipation emulating microwave networks $\Gamma _{1p}$ (blue dotted line) and $\Gamma _{2p}$ (red solid line). In the calculations performed for graphs $\Gamma _{1p}$ and $\Gamma _{2p}$, Eqs. (7) and (5), and (8) and (5) were applied, respectively.

In this article, we study graphs and microwave networks characterized by time-reversal invariance. Microwave realizations of isoscattering non-isospectral graphs $\Gamma _1$ and $\Gamma _2$ with Kirchhoff-Neumann boundary conditions are shown in Fig. 3. The proper simulation of quantum graphs by microwave networks requires fulfilling the Kirchoff-Neumann boundary conditions with high accuracy. This was the case in the presented experiment. For example, the reflection and transmission amplitudes of the 4-way junctions used in the experiment were in the range of $0.50 \pm 0.01$, which were close to the theoretical values of 0.5. The leads $\mathscr {L}^{\infty }_1$ and $\mathscr {L}^{\infty }_2$ in the microwave networks are connected in the contact vertices defined in Fig. 1. To obtain the one-port scattering matrix $S(k)=|S(k)|\exp {(i\varphi )}$, where |*S*(*k*)| and $\varphi$ are the amplitude and the phase of the scattering matrix, the scattering matrix was evaluated in the frequency range $\nu = 0.01-1$ GHz (see Figs. 4 and 5). The wave vector *k* expressed in terms of the frequency $\nu$ is defined as $k=\frac{2\pi \nu }{c}$, where *c* is the speed of light in vacuum. The connections of the VNA to microwave networks $\Gamma _1$ and $\Gamma _2$ (see Fig. 3) are equivalent to attaching of the infinite leads $\mathscr {L}^{\infty }_1$ and $\mathscr {L}^{\infty }_2$.

In Fig. 4a, b we show the amplitudes |*S*(*k*)| and the phases $\varphi$ of the scattering matrices *S*(*k*) of the experimentally studied isoscattering non-isospectral microwave networks $\Gamma _1$ (blue broken line) and $\Gamma _2$ (red solid line) with Kirchhoff-Neumann boundary conditions in the frequency range 0.01-1 GHz. In Fig. 4a, the theoretical positions of the resonances $\sigma _{\Gamma _1}(k)$ and $\sigma _{\Gamma _2}(k)$ are marked by blue circles and red crosses for graphs $\Gamma _1$ and $\Gamma _2$, respectively. Singular resonances of the dissipation-free graph $\Gamma _{2}$, $k_3=\frac{\pi }{2\ell } \simeq 4.19$ $\frac{1}{m}$ and $k_8=\frac{3\pi }{2\ell } \simeq 12.58$ $\frac{1}{m}$, are indicated by black arrows.

The amplitudes and phases of the scattering matrices for both networks are in almost perfect agreement. Without prior knowledge that both graphs are non-isospectral, one might erroneously conclude that they are isospectral. This would be a likely interpretation of the experimental results.

As discussed previously, to demonstrate experimentally that the graphs $\Gamma _1$ and $\Gamma _2$ are non-isospectral, minor alterations to their structure are required and because of that the parameter $\xi$ was introduced (see Fig. 2).

Panels (a) and (b) in Fig. 5 show the scattering matrix amplitudes |*S*(*k*)| and the phases $\varphi$ of the experimentally studied microwave networks $\Gamma _{1p}$ (blue dotted line) and $\Gamma _{2p}$ (red solid line) with Kirchhoff-Neumann boundary conditions in the frequency range 0.01-1 GHz. The networks were characterized by the same total length $L=6l=2.247 \pm 0.003$ m and the parameter $\xi$ was chosen to be 0.02 m.

Figure 5 shows that modifying the graphs results in a deviation from isoscattering, thereby unveiling distinct spectral structures and phase dependence on the *k* vector. Though, the spectra of graphs are generally degenerate, modifying the largest loop with a circumference of 4*l* in the network $\Gamma _{2p}$ results in singular resonances around $k_3 \simeq 4.19$ $\frac{1}{m}$ and $k_8 \simeq 12.60$ $\frac{1}{m}$ for frequencies below 0.75 GHz. The resonances are indicated by the black arrows. Their appearance clearly shows that both networks are non-isospectral.

As expected, despite the presence of absorption, the positions of the resonances are very close to the theoretically predicted values: $k_3 \simeq 4.19$ $\frac{1}{m}$ and $k_8 \simeq 12.58$ $\frac{1}{m}$ obtained for the dissipation-free graph $\Gamma _2$.

It should be noted that the scattering matrix amplitude, |*S*(*k*)|, for the parameter, $\xi =0.02$ m, also revealed other resonances, which can be explained by the symmetry breaking in the graphs $\Gamma _1$ and $\Gamma _2$ in Fig. 1a, b. The symmetry axes passing through the vertices of graphs $\Gamma _1$ and $\Gamma _2$ are destroyed, causing the M-functions to depend strongly on the parameter $\xi$. Therefore, as outlined in the following section (see Fig. 5c, d), the theoretical results align closely with the experimental ones.

In Figs. 4c, d and 5c, d the absolute value |*S*(*k*)| and the phase $\varphi$ of the scattering matrices *S*(*k*) calculated for the quantum graphs with dissipation simulating microwave networks are displayed. In the calculations provided for graphs $\Gamma _1$ and $\Gamma _2$, dissipative M-functions $M_{\Gamma _1}(k)$ and $M_{\Gamma _2}(k)$ were applied in accordance to Eqs. (11) and (5). In the case of the graphs with perturbation $\Gamma _{1p}$ and $\Gamma _{2p}$, Fig. 5c, d, Eqs. (7), (8) and (5) were applied. Dissipation was included in the calculations by employing the following substitution of the wave vector^31^ in the aforementioned equations: $k \mapsto k + i \beta \sqrt{k}$ with $\beta =0.0098 \hbox { m}^{-1/2}$.

The theoretical and experimental results demonstrate a high degree of agreement, thus substantiating the effectiveness of the experimental method of simulating quantum graphs and demonstrating that both graphs are non-isospectral.

## Summary

The M-function formalism was applied to identify two non-isospectral isoscattering graphs. The theoretical predictions were validated through experimental studies of microwave networks. By altering the lengths of the edges of the microwave networks and analyzing the resonances of the networks, it was demonstrated that both graphs, despite being isoscattering, were indeed non-isospectral. The present analysis reveals the potential surprises that can be encountered during the interpretation of scattering matrix measurements. This is of particular importance because such measurements are frequently the sole available tool for the identification of resonances in complex systems.

## Data Availability

The data that support results presented in this paper and other findings of this study are available from the correspondingauthors upon reasonable request.
